# Physiologically-based pharmacokinetic modeling to predict drug-drug interaction of enzalutamide with combined P-gp and CYP3A substrates

**DOI:** 10.1007/s10928-023-09867-7

**Published:** 2023-06-21

**Authors:** Yukio Otsuka, Srinivasu Poondru, Peter L. Bonate, Rachel H. Rose, Masoud Jamei, Fumihiko Ushigome, Tsuyoshi Minematsu

**Affiliations:** 1 Clinical Pharmacology and Exploratory Development, Astellas Pharma Inc., 2-5-1, Nihonbashi-honcho, Chuo-ku, Tokyo, 103-8411 Japan; 2 Clinical Pharmacology and Exploratory Development, Astellas Pharma Global Development Inc., Northbrook, IL USA; 3 Simcyp Division, Certara UK, Sheffield, UK; 4 Applied Research and Operations, Astellas Pharma Inc., Ibaraki, Japan; 5 Immuno-oncology, Astellas Pharma Inc., Ibaraki, Japan

**Keywords:** Physiologically-based pharmacokinetics (PBPK), Drug-drug interaction (DDI), Enzalutamide, Apixaban, Rivaroxaban, P-glycoprotein (P-gp)

## Abstract

Enzalutamide is known to strongly induce cytochrome P450 3A4 (CYP3A4). Furthermore, enzalutamide showed induction and inhibition of P-glycoprotein (P-gp) in in vitro studies. A clinical drug-drug interaction (DDI) study between enzalutamide and digoxin, a typical P-gp substrate, suggested enzalutamide has weak inhibitory effect on P-gp substrates. Direct oral anticoagulants (DOACs), such as apixaban and rivaroxaban, are dual substrates of CYP3A4 and P-gp, and hence it is recommended to avoid co-administration of these DOACs with combined P-gp and strong CYP3A inducers. Enzalutamide’s net effect on P-gp and CYP3A for apixaban and rivaroxaban plasma exposures is of interest to physicians who treat patients for venous thromboembolism with prostate cancer. Accordingly, a physiologically-based pharmacokinetic (PBPK) analysis was performed to predict the magnitude of DDI on apixaban and rivaroxaban exposures in the presence of 160 mg once-daily dosing of enzalutamide. The PBPK models of enzalutamide and M2, a major metabolite of enzalutamide which also has potential to induce CYP3A and P-gp and inhibit P-gp, were developed and verified as perpetrators of CYP3A-and P-gp-mediated interaction. Simulation results predicted a 31% decrease in AUC and no change in C_max_ for apixaban and a 45% decrease in AUC and a 25% decrease in C_max_ for rivaroxaban when 160 mg multiple doses of enzalutamide were co-administered. In summary, enzalutamide is considered to decrease apixaban and rivaroxaban exposure through the combined effects of CYP3A induction and net P-gp inhibition. Concurrent use of these drugs warrants careful monitoring for efficacy and safety.

**Supplementary Information:**

The online version contains supplementary material available at 10.1007/s10928-023-09867-7.

## Introduction

Enzalutamide (MDV3100) is an androgen receptor (AR) inhibitor that targets the AR signaling pathway. Enzalutamide was approved in the US in 2012 under the tradename XTANDI^®^ for the treatment of patients with metastatic castration-resistant prostate cancer (CRPC) who have previously received docetaxel. The indication for XTANDI was subsequently expanded to the treatment of metastatic CRPC and later to the treatment of CRPC. XTANDI was also approved in 2019 in the US for the treatment of metastatic castration-sensitive prostate cancer (mCSPC; also referred to as metastatic hormone-sensitive prostate cancer [mHSPC]). Worldwide, enzalutamide has been approved for the treatment of metastatic CRPC in more than 100 countries.

Oral absorption of enzalutamide, whether administered as single or multiple doses, is rapid and dose-independent. Peak concentrations of enzalutamide are generally achieved 1 to 2 h postdose in both patients and healthy subjects [1]. Enzalutamide is well absorbed (estimated bioavailability based on mass balance data ≥ 84%), an expected finding for a low extraction ratio drug that displays high permeability and is not a substrate for P-glycoprotein (P-gp) or breast cancer resistance protein (BCRP). A high-fat meal slows the rate of enzalutamide absorption, but the extent of absorption is unaffected. Enzalutamide has been administered without regard to meals in clinical studies in patients, including pivotal phase three studies. In vitro and in vivo protein binding data showed that enzalutamide is 97% to 98% bound to plasma proteins, primarily albumin. *N*-desmethyl enzalutamide (M2), the active metabolite of enzalutamide, is 95% to 97% bound to plasma proteins. In vitro studies show that enzalutamide is metabolized by cytochrome P450 2C8 (CYP2C8) and CYP3A4/5, both of which play a role in the formation of M2. In addition, an in vitro study showed that human carboxylesterase 1 (hCES1) is capable of catalyzing the formation of the carboxylic acid metabolite (M1) from enzalutamide and M2. Following oral administration of ^14^C-enzalutamide to healthy male subjects, 85% of the dose was recovered through day 77 postdose: 71% was recovered in urine (primarily as M1, with trace amounts of enzalutamide and M2) and 14% was recovered in feces (0.39% as enzalutamide) [1]. Based on this information, renal excretion is considered a minor elimination pathway for unchanged parent enzalutamide and M2. With assuming all components, including M1, in metabolite profiling which possibly generated from M2 were formed from M2, M2 formation accounted for 63% of dosed enzalutamide.

The potential for enzalutamide to affect the pharmacokinetics of other drugs was assessed in in vivo phenotypic cocktail drug-drug interaction (DDI) studies. At steady state, enzalutamide is a strong CYP3A4 inducer and a moderate CYP2C9 and CYP2C19 inducer [2]. Enzalutamide did not cause clinically meaningful changes in exposure to the CYP1A2, CYP2C8, or CYP2D6 substrates. In a clinical DDI study, enzalutamide increased the AUC of digoxin, a typical P-gp substrate, by 29%, indicating the net effect of enzalutamide on P-gp is inhibition-based [3]. Clinical DDI study results with rosuvastatin, a typical BCRP substrate, showed that enzalutamide did not change the plasma exposure of rosuvastatin.

In 9% of prostate cancer patients, venous thromboembolism (VTE) with high mortality risk was reported [4]. For the treatment of VTE, anticoagulation is essential in preventing thrombosis. Direct oral anticoagulants (DOACs) as first-line agents for the treatment of VTE with cancer are recommended in the guidelines issued by the American College of Chest Physicians [5] and the American Society of Hematology [6]. Drug interactions during DOAC therapy can dramatically alter their efficacy and safety profiles. Apixaban and rivaroxaban are two DOACs, both of which are dual CYP3A and P-gp substrates. Hence, drug interactions with enzalutamide are one of the concerns in VTE treatment in prostate cancer patients [7].

This study aimed to predict the magnitude of CYP3A- and P-gp-mediated induction and inhibitory effects of enzalutamide and M2 on apixaban and rivaroxaban. The in vitro ability of enzalutamide to induce CYP enzymes and P-gp, and to inhibit P-gp, was investigated. Both in vitro and in vivo clinical data were then integrated into a physiologically-based pharmacokinetic (PBPK) model to quantitatively estimate the magnitude of exposure change in apixaban and rivaroxaban when enzalutamide is concomitantly administered at clinically relevant doses.

## Methods

### In vitro CYP and P-gp induction study

The in vitro potential of enzalutamide and two major metabolites, M1 and M2, to induce CYP enzymes and P-gp was investigated with freshly isolated human hepatocytes. Hepatocytes were cultured according to previously described methods [8]. Enzalutamide, M1, or M2 were treated with three concentrations (1, 10, or 100 µmol/L). As positive controls, 50 µmol/L omeprazole, 750 µmol/L phenobarbital, or 10 µmol/L rifampicin were tested. Approximately 24 h after the last treatment, hepatocytes were lysed in TRIzol^®^ reagent and total RNA was phase-extracted from the cell lysates according to the TRIzol procedure. Single-stranded cDNA was prepared from RNA with the RT Master Mix using the AB 7900HT Fast Real Time PCR System thermocycling program. Quantitative RT-PCR was carried out in triplicate. Relative quantification measures the change in mRNA expression in a test sample relative to that in a control sample (e.g., DMSO). This method assumes that the efficiency of the target amplification and the efficiency of the endogenous control amplification are approximately equal.

The maximal fold induction (Ind_max_) and half concentration to reach Ind_max_ (IndC_50_) were determined based on the following equation:

$$\mathrm{Fold\, induction}=1+\frac{\left({\mathrm{Ind}}_{\mathrm{max}}-1\right)\times {\mathrm{C}}_{\mathrm{t}}}{{\mathrm{IndC}}_{50}+{\mathrm{C}}_{\mathrm{t}}}$$

where C_t_ is the test drug concentration.

### In vitro P-gp inhibition study

MDR1-expressing cells (porcine kidney epithelial LLC-PK1 cells transfected with human MDR1 cDNA) was used. The MDR1-expressing cells were seeded at a density of 4 × 10^4^ cells/insert in plates and were incubated in a CO_2_ incubator (37 °C, CO_2_: 5%) for 7 to 9 days to prepare cell monolayers. After the cells were pre-incubated at 37 °C for 1 h, in half of the plates, the medium in the insert chamber (i.e., the apical side) was aspirated and replaced with 100 μL of test solutions, which incorporated 1 µmol/L of ^3^H-digoxin and enzalutamide (0, 0.3, 1, 3, 10, 30, and 50 μmol/L) or M2 (0, 0.1, 0.3, 1, 3, 10, and 25 μmol/L); in the other half of the plates, the media in the well (i.e., the basal side) were aspirated and replaced with 600 μL of test solution. After incubation for 4 h at 37 °C, a 70-μL aliquot of the incubation solutions was collected as the analytical sample from the opposite compartment of that spiked with test solutions (receiver compartment). The collected samples were immediately combined with 10 mL of scintillator, and the radioactivity was counted using the LSC. Permeability coefficient (P_app_) of ^3^H-digoxin was calculated using the following equation:

$$\text{P}_\text{app} \,\left( {\text{cm}/\sec } \right)  = [\text{dQ/dt]}/{\text{A/C}_{0}}$$

where dQ/dt is the transport rate (dpm/sec); A is the membrane area (cm^2^); C_0_ is the initial concentration (dpm/mL). The ratio of permeability coefficient (P_app_ ratio) was calculated from the basal to apical P_app_ and apical to basal P_app_ according to the following equation:

$${\mathrm{P}}_{\mathrm{app}}\,\mathrm{ ratio}=\frac{\mathrm{Basal\, to\, apical }\,{\mathrm{P}}_{\mathrm{app}}}{\mathrm{Apical\, to\, basal }\,{\mathrm{P}}_{\mathrm{app}}}$$

Furthermore, the ratios of the P_app_ ratios in MDR1-expressing cells to the P_app_ ratios in control cells were calculated as corrected P_app_ ratio according to the following equation:

$$\mathrm{Corrected }\,{\mathrm{P}}_{\mathrm{app}}\,\mathrm{ ratio}= \frac{{\mathrm{P}}_{\mathrm{app}}\,\mathrm{ ratio\,of\,the\,MDR}1\,\mathrm{ expressing\,cells}}{{\mathrm{P}}_{\mathrm{app}}\,\mathrm{ ratio\,of\,the\,control\,cells}}$$

The % of control was calculated from the corrected P_app_ ratio according to the following equation:

$$\text{\% \,of\,control} = \frac{{\text{Corrected}\,{\text{P}_{\text{app}} \,\text{ratio\,in\,the\,absence\,or\,presence\,of\,inhibitors}}}}{{\text{Corrected}\,{\text{P}_{\text{app}} \,\text{ratio\,in\,the\,absence\,of\,inhibitors}}}}\,{\times}\,100$$

The IC_50_ value was calculated by the least squares method from the relationship between enzalutamide or M2 concentration and the % of control according to the following equation:

$$\frac{{\mathrm{R}}_{\mathrm{i}}}{{\mathrm{R}}_{\mathrm{a}}}=(1-\frac{{\mathrm{I}}_{\mathrm{max}}\times {\mathrm{I}}^{\mathrm{C}}}{{\mathrm{I}}^{\mathrm{C}}+{{\mathrm{IC}}_{50}}^{\mathrm{C}}})\times 100$$

where R_i_ and R_a_ are the corrected P_app_ ratio in the presence and absence of enzalutamide or M2, respectively; I_max_ is the maximum inhibitory effect; I is the concentration of enzalutamide or M2; and C is the Hill factor.

### PBPK model development and verification

The PBPK models for enzalutamide and M2 were developed within the Simcyp Simulator version 19 (Certara UK Limited, Sheffield, UK). The advanced dissolution, absorption, and metabolism (ADAM) model for absorption and the full PBPK model for distribution were used for enzalutamide, and the minimum PBPK distribution model was used for M2.

Many of the physicochemical properties and plasma protein binding data were obtained internally and also referenced from FDA documents [9], which are available from the FDA website. The cLog P of M2 was calculated in silico (ACD/Labs percepta 14.3.0, Build 3063, Advanced Chemistry Development Inc., Toronto, Canada). The enzalutamide model was constructed for a liquid-filled capsule formulation. Accordingly, the solution formulation option in the Simcyp model was used. Permeability (P_eff,man_) was predicted from Caco-2 passive permeability data, including calibration of enzalutamide permeability to the measured permeability of the propranolol as a reference. The volume of distribution was predicted using the Simcyp built-in methods (Method 2 for enzalutamide and Method 1 for M2) with slight modification in enzalutamide using the K_p_ scalar to obtain clinically observed volume of distribution [9]. An in vitro study suggested enzalutamide was metabolized by CYP3A4 and CYP2C8 forming M2 [2]. From the clinical mass balance and metabolite profiling data, 63% of dosed enzalutamide was metabolized to M2. The contribution of CYP2C8 and CYP3A4 to the formation of M2 was estimated from a clinical DDI study result using gemfibrozil, a strong CYP2C8 inhibitor, as the perpetrator. The elimination pathway of M2 is not known; however, concomitant administration of itraconazole, a strong CYP3A4 inhibitor, increased AUC_inf_ of M2 in the clinical DDI study [2], indicating possible contribution of CYP3A4 to the elimination of M2. The CYP3A4 contribution to M2 elimination was determined from exposure after multiple-dose administration of enzalutamide, which was possibly affected with autoinduction of CYP3A4. The ability of the enzalutamide and M2 PBPK models to predict CYP3A induction was confirmed using a DDI simulation with midazolam as the victim. The net effect of enzalutamide on P-gp was investigated in a clinical DDI study using digoxin as a typical P-gp substrate [3]. Enzalutamide inhibition and induction effects to P-gp were optimized based on the results from a clinical DDI study.

The PBPK models of CYP2C8 inhibitor (gemfibrozil), CYP3A4 substrate (midazolam), and P-gp substrate (digoxin) were used from the Simcyp software library with models that were verified for their intended purpose.

### Application to DDI simulation

The DDI prediction of enzalutamide with apixaban and rivaroxaban (CYP3A and P-gp substrates) in a cancer population was performed in 100 virtual subjects. The PBPK models for apixaban and rivaroxaban were previously developed and verified for CYP3A- and P-gp-mediated interaction [10]. Two models for rivaroxaban, one considered OAT3 involvement in renal elimination (Model 1) and one did not (Model 2), were reported. Accordingly, the rivaroxaban DDI was investigated for both these models. Sensitivity analysis was conducted on the influence of intestinal P-gp activities of apixaban and rivaroxaban on the DDI with enzalutamide. P-gp relative activity factor/relative expression factor (RAF/REF) is used to scale in vitro to in vivo activity by multiplying RAF/REF to in vitro P-gp intrinsic clearance (CL_int,p-gp_; apixaban) or maximal rate of P-gp-mediated drug transport (J_max_; rivaroxaban). During the model development process, intestinal P-gp activity was adjusted by modifying the P-gp RAF/REF values. Accordingly, a sensitivity analysis was performed to assess the impact of these changes on the predicted magnitude of the DDI.

## Results

### In vitro CYP and P-gp induction and P-gp inhibition studies

In the CYP and P-gp induction study, there was a tendency that the increases in mRNA expression were highest following treatment with enzalutamide 10 μmol/L not the highest drug concentration tested, 100 μmol/L (Table 1).

**Table 1:** The effects of treating culture human hepatocytes with enzalutamide, M2, or prototypical inducers on CYP and P-gp mRNA levels

Treatment	Conc. (µmol/L)	Fold increase						
		CYP1A2	CYP2B6	CYP2C8	CYP2C9	CYP2C19	CYP3A4	P-gp
Enzalutamide	1	1.14 ± 0.53	1.50 ± 0.45	2.14 ± 0.74	1.41 ± 0.27	1.07 ± 0.15	2.24 ± 0.82	1.17 ± 0.07
Enzalutamide	10	1.06 ± 0.23	3.36 ± 0.72	10.9 ± 5.2	3.28 ± 0.71	1.92 ± 1.22	6.90 ± 2.25	2.16 ± 0.29
Enzalutamide	100†	0.333	2.34	3.12	1.10	1.26	4.62	1.74
Ind_max_		–	–	–	–	–	5.9	–
IndC_50_		–	–	–	–	–	1.5	–
M1	1	0.697 ± 0.260	0.724 ± 0.316	1.11 ± 0.53	0.853 ± 0.361	0.855 ± 0.246	0.832 ± 0.417	0.885 ± 0.553
M1	10	0.883 ± 0.303	1.03 ± 0.43	1.44 ± 0.50	0.888 ± 0.172	1.06 ± 0.10	1.17 ± 0.46	1.46 ± 0.79
M1	100	1.01 ± 0.59	0.848 ± 0.337	2.35 ± 1.66	1.52 ± 0.63	1.55 ± 0.80	1.83 ± 0.98	1.32 ± 0.25
M2	1	0.561 ± 0.100	0.705 ± 0.208	1.73 ± 1.10	1.00 ± 0.27	0.872 ± 0.205	1.42 ± 0.31	0.894 ± 0.513
M2	10	0.960 ± 0.279	1.96 ± 0.14	7.40 ± 3.26	2.38 ± 0.48	1.18 ± 0.46	5.30 ± 2.77	1.66 ± 0.61
M2	100†	0.842	1.80	8.93	1.50	2.12	4.32	1.63
Ind_max_		–	–	–	–		5.1	–
IndC_50_		–	–	–	–		2.5	–
Omeprazole	50	46.4 ± 7.2	–	–	–	–	–	2.38 ± 1.68
Phenobarbital	750	–	6.90 ± 3.66	–	–	–	–	2.61 ± 0.59
Rifampicin	10	–	–	14.0 ± 5.6	3.64 ± 0.47	3.80 ± 3.61	8.05 ± 4.10	3.60 ± 2.30

Data were expressed as mean ± SD from three lots of hepatocyte

^†^data for 100 µmol/L were obtained from two lots of hepatocyte

Of the CYP isozymes tested, CYP3A4 showed the greatest increase following enzalutamide or M2 treatment, with up to a 6.90-fold increase (95.9% of positive control) in mRNA levels being observed, on average. In contrast, CYP1A2 did not show an increase in mRNA levels, while the other CYP isozymes (CYP2B6, CYP2C8, CYP2C9, and CYP2C19) showed some small increases in mRNA levels. For P-gp mRNA expression levels, treatment with 10 µmol/L enzalutamide or M2 caused a 2.16- and 1.66-fold increase, respectively, on average. The Ind_max_ and IndC_50_ for CYP3A4 were determined as 5.9-fold and 1.5 µmol/L, respectively, for enzalutamide and 5.1-fold and 2.5 µmol/L, respectively, for M2. When input to the Simcyp simulator, Ind_max_ was calibrated with positive control, rifampicin, and the calibrated values were 11.43 and 9.72 for enzalutamide and M2, respectively. M1 showed only minor effects on CYP enzymes and P-gp mRNA expression levels.

In the P-gp inhibition study, the corrected P_app_ ratio of ^3^H-digoxin across MDR1-expressing cells was decreased when co-incubated with increasing enzalutamide or M2 concentrations (Table 2).

**Table 2:** Inhibitory effect of enzalutamide on ^3^H-digoxin (1 µmol/L) permeation across control and MDR1-expressing cell monolayers

Additive	Concentration (µmol/L)	Control cells			MDR1-expressing cells			Corrected P_app_ ratio	IC_50_ (µmol/L)
		P_app_ (× 10^–6^ cm/sec)		P_app_ ratio	P_app_ (× 10^–6^ cm/sec)		P_app_ ratio		
		A to B	B to A		A to B	B to A			
Enzalutamide	0	1.73 ± 0.73	1.66 ± 0.06	1.0	0.641 ± 0.028	12.8 ± 0.6	20.0	20.0	1.67
	0.3	1.39 ± 0.20	1.85 ± 0.31	1.3	0.725 ± 0.043	12.4 ± 0.8	17.1	13.2	
	1	1.47 ± 0.14	1.94 ± 0.13	1.3	0.851 ± 0.081	13.4 ± 0.4	15.7	12.1	
	3	1.68 ± 0.13	2.02 ± 0.10	1.2	1.25 ± 0.26	12.1 ± 0.9	9.7	8.1	
	10	2.09 ± 0.07	2.20 ± 0.10	1.1	1.41 ± 0.08	11.0 ± 0.6	7.8	7.1	
	30	2.42 ± 0.24	2.56 ± 0.19	1.1	2.11 ± 0.09	7.85 ± 0.28	3.7	3.4	
	50	2.22 ± 0.21	2.63 ± 0.32	1.2	2.73 ± 0.12	7.22 ± 0.18	2.6	2.2	
M1	0	1.39 ± 0.09	1.66 ± 0.23	1.2	0.683 ± 0.105	15.6 ± 1.2	22.8	19.0	–
	0.3	1.22 ± 0.06	1.62 ± 0.22	1.3	0.807 ± 0.032	13.8 ± 1.1	17.1	13.2	
	1	1.31 ± 0.06	1.62 ± 0.03	1.2	0.670 ± 0.029	14.0 ± 1.2	20.9	17.4	
	3	1.39 ± 0.05	1.44 ± 0.13	1.0	0.725 ± 0.011	13.9 ± 0.4	19.2	19.2	
	10	1.36 ± 0.11	1.36 ± 0.12	1.0	0.626 ± 0.033	13.6 ± 1.0	21.7	21.7	
	30	1.43 ± 0.06	1.46 ± 0.13	1.0	0.715 ± 0.045	13.1 ± 0.4	18.3	18.3	
	80	1.43 ± 0.10	1.47 ± 0.13	1.0	0.623 ± 0.035	13.3 ± 0.9	21.3	21.3	
M2	0	1.39 ± 0.09	1.66 ± 0.23	1.2	0.683 ± 0.105	15.6 ± 1.2	22.8	19.0	1.09
	0.1	1.45 ± 0.04	1.54 ± 0.08	1.1	0.727 ± 0.088	14.1 ± 0.2	19.4	17.6	
	0.3	1.83 ± 0.09	2.03 ± 0.15	1.1	0.723 ± 0.170	13.4 ± 1.0	18.5	16.8	
	1	1.38 ± 0.13	1.46 ± 0.10	1.1	1.01 ± 0.02	12.1 ± 0.6	12.0	10.9	
	3	1.54 ± 0.11	1.64 ± 0.12	1.1	1.89 ± 0.02	11.0 ± 0.8	5.8	5.3	
	10	1.51 ± 0.11	1.66 ± 0.07	1.1	2.89 ± 0.17	8.40 ± 0.38	2.9	2.6	
	25	1.68 ± 0.05	1.73 ± 0.11	1.0	2.83 ± 0.12	6.19 ± 0.25	2.2	2.2	

A to B: apical to basal, B to A: basal to apical

The IC_50_ values were estimated to be 1.67 and 1.09 µmol/L for enzalutamide and M2, respectively. The concentration of digoxin was set smaller than its K_m_ value and determined IC_50_ values were regarded as inhibition constant (K_i_) in the DDI simulation. M1 did not inhibit P-gp-mediated ^3^H-digoxin transport.

### PBPK model development and verification of enzalutamide and M2

Based on the clinical DDI study result with gemfibrozil, the contribution of CYP2C8 on enzalutamide elimination was estimated to be 87%, with the remaining percent assigned to CYP3A4-mediated metabolism. Further, setting 67% of CYP2C8 CL_int_ to M2 formation and the remaining 33% to other pathways allowed recovery of the M2 AUC ratio. To maintain conversion of enzalutamide to M2 at 63%, the fraction of CYP3A4 CL_int_ contributing to M2 formation was set at 39% and the remaining 61% of CYP3A4 CL_int_ was assigned to other pathways. In the assignment of M2 elimination pathway, CYP3A4 contribution of 9% resulted in recovery of M2 exposure after multiple doses of enzalutamide. For the enzalutamide model, the CL_int_ of each pathway was calculated from the CL_po_ of 0.60 L/h observed in a food effect study using the retrograde calculation method in the Simcyp simulator (Table 3).

**Table 3:** Parameters for enzalutamide and M2 PBPK models

	Enzalutamide		N-Desmethyl enzalutamide (M2)	
Parameters	Value	Assumption(s) and references	Value	Assumption(s) and references
Physicochemical properties and blood binding				
Compound type	Neutral	FDA CP review	Neutral	
Molecular weight	464.44	FDA CP review	450.41	FDA CP review
Log P	2.98	FDA CP review	2.11	In silico calculation with ACD/Labs
B/P ratio	0.55	FDA CP review	0.55	Assumed
f_u_	0.0244	FDA CP review	0.0467	FDA CP review
Plasma binding protein	HSA	FDA CP review	HSA	FDA CP review
Absorption				
Absorption model	ADAM		–	
f_u,gut_	0.0244	Assumed to same to f_u_	0.0467	Assumed to same to f_u_
P_app_ (10^–6^ cm/s) Caco-2 passive permeability (pH6.5:7.5)	31	Internal data	–	
Reference P_app_ (10^–6^ cm/s) Caco-2 passive permeability of propranolol	14.8	Internal data	–	
P_eff,man_ (10^–4^ cm/s)	5.15	Calculated	–	
Formulation	Solution	Assumed	–	
Distribution				
Distribution model	Full PBPK		Minimal PBPK	
Prediction method of V_ss_	Method 2		Method 1	
V_ss_ (L/kg)	0.967	[1]	0.48	Estimated
K_p_ scalar	0.933	Optimized	1	
Elimination				
CL_R_ (L/h)	0	[1]	0	[1]
CYP2C8 CL_int_ for M2 formation (mL/min/pmol enzyme)	0.1518	See text	–	
CYP2C8 CL_int_ for other pathway (mL/min/pmol enzyme)	0.07475	See text	–	
CYP3A4 CL_int_ for M2 formation (mL/min/pmol enzyme)	0.002312	See text	0.001075	See text
CYP3A4 CL_int_ for other pathway (mL/min/pmol enzyme)	0.003617	See text	–	
CL_int,HLM_ (μL/min/mg protein)			1.399	See text
Interaction				
K_i_ on CYP3A4 (μmol/L)	42	Internal data	–	
K_i_ on CYP3A5 (μmol/L)	42	Internal data	–	
Ind_max_ on CYP3A4	11.43	See text	9.72	See text
IndC_50_ on CYP3A4 (μmol/L)	1.5	See text	2.5	See text
Ind_max_ on CYP3A5	11.43	Assumed to be same to CYP3A4	9.72	Assumed to be same to CYP3A4
IndC_50_ on CYP3A5 (μmol/L)	1.5	Assumed to be same to CYP3A4	2.5	Assumed to be same to CYP3A4
K_i_ on P-gp (μmol/L)	1.67	See text	1.09	See text
K_i_ on OAT3 (μmol/L)	15.1	Internal data	11.5	Internal data

**Fig. 1:**
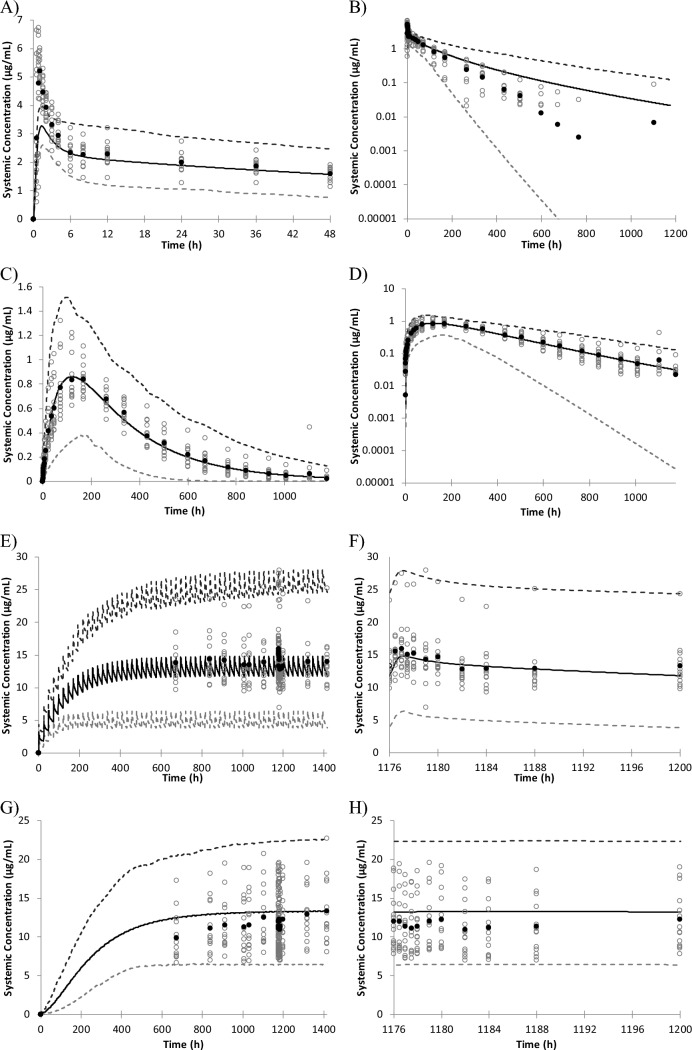
Observed and simulated plasma concentration–time profiles of enzalutamide and M2: (**A**, **B**) enzalutamide data after single 160 mg dose in linear and semi-log scales; (**C**, **D**) M2 data after single 160 mg dose in linear and semi-log scales; (**E**, **F**) enzalutamide data after multiple 160 mg doses in full-time scale and extracting 1176 to 1200 h; (**G**, **H**) M2 data after multiple 160 mg doses in full-time scale and extracting 1176 to 1200 h. The data shown are simulated mean (solid line), simulated 5th and 95th percentiles (dashed lines), observed mean (filled circles), and observed individual (open circles)

The predictability of plasma concentration–time profiles (Fig. 1) and PK parameters (Table S1, Table S2) was confirmed after single and multiple oral administration of enzalutamide in clinical studies. The datasets are independent from enzalutamide model development, except for M2 concentration data after multiple doses were used for the determination of CYP3A4 contribution in hepatic metabolism of M2. The developed enzalutamide and M2 models showed good reproducibility of the plasma concentration–time profiles when 160 mg enzalutamide was administered once, or multiple times (Fig. 1).

**Fig. 2:**
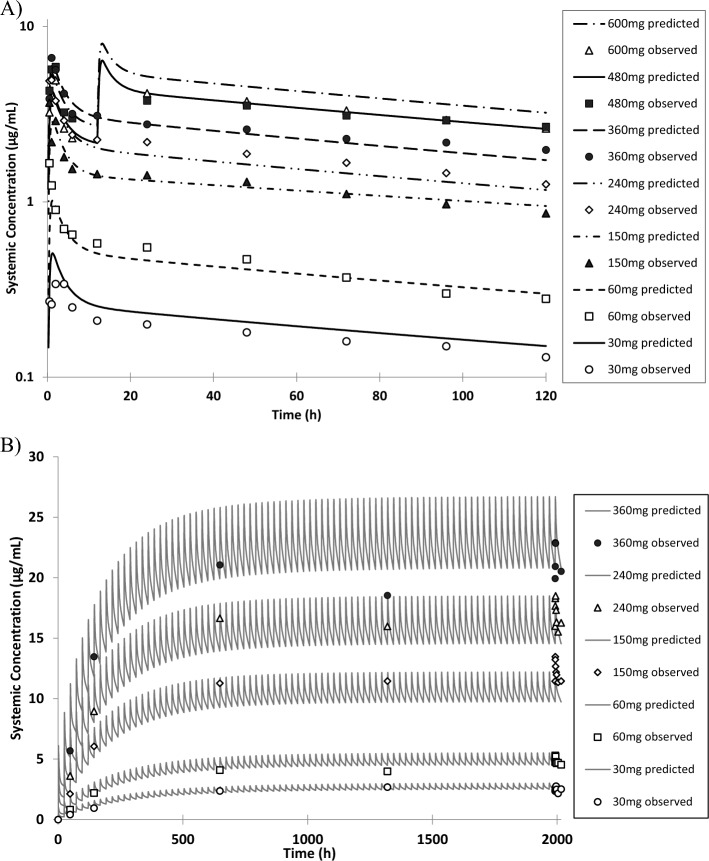
Observed and simulated plasma concentration–time profiles of enzalutamide after (**A**) single and (**B**) multiple doses at several dose levels. The data shown are simulated mean (lines) and observed mean (markers)

Further, the model capability to simulate enzalutamide plasma concentrations after single- and multiple-dose administration at several dose levels was confirmed (Fig. 2).

Due to autoinduction of CYP3A4, both the capability of the model to capture multiple-dose clinical data and the DDI simulation result with midazolam confirmed the developed models were verified for modelling CYP3A induction effects in clinical aplications (Table 4).

**Table 4:** Observed and simulated AUC and C_max_ ratios of midazolam and digoxin in presence or absence of multiple 160 mg doses of enzalutamide

		Without enzalutamide		With enzalutamide		AUC ratio	C_max_ ratio
		AUC (ng·h/mL)	C_max_ (ng/mL)	AUC (ng·h/mL)	C_max_ (ng/mL)		
Midazolam							
Observed		32.0 (12.8)	9.98 (3.17)	4.22 (0.784)	2.29 (0.868)	0.14 (0.12–0.17)	0.23 (0.20–0.27)
Simulated		34.4 (24.0)	9.56 (5.93)	4.03 (2.84)	1.91 (1.32)	0.12 (0.11–0.12)	0.19 (0.18–0.20)
Criteria		–	–	–	–	0.07–0.27	0.13–0.42
Digoxin							
Observed		22.1 (4.10)	1.45 (0.314)	28.7 (5.79)	1.69 (0.384)	1.29 (1.21–1.38)	1.17 (1.06–1.29)
Simulated	FI = 1.0	20.0 (6.89)	1.12 (0.322)	25.1 (6.81)	1.54 (0.367)	1.29 (1.19–1.40)	1.38 (1.30–1.47)
	FI = 2.0	20.0 (6.89)	1.12 (0.322)	21.1 (7.11)	1.23 (0.362)	1.06 (0.964–1.16)	1.09 (1.02–1.17)
	FI = 3.5	20.0 (6.89)	1.12 (0.322)	16.5 (7.10)	0.953 (0.341)	0.789 (0.709–0.879)	0.828 (0.767–0.893)
Criteria	–	–	–	–	–	0.91–1.83	0.86–1.59

Data were expressed with mean (SD) for AUC and C_max_ and geometric mean ratio (90% CI) for AUC and C_max_ ratios

Criteria were calculated based on the predictive measure proposed by Guest et al. [20]

**Fig. 3:**
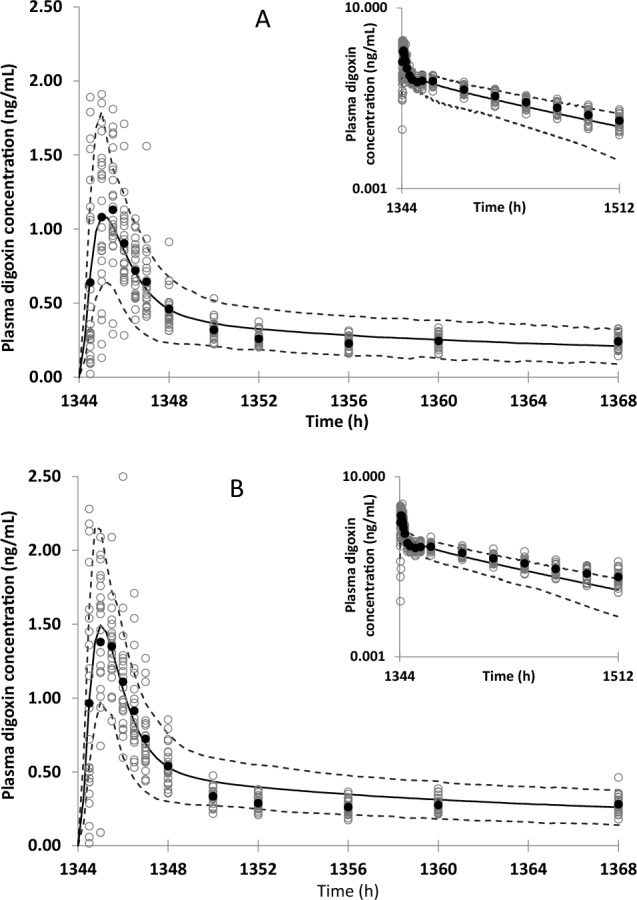
Observed and simulated plasma concentration–time profiles of digoxin in (**A**) absence and (**B**) presence of enzalutamide. The data shown are simulated mean (solid line), simulated 5th and 95th percentiles (dashed lines), observed mean (filled circles), and observed individual (open circles)

Furthermore, the enzalutamide and M2 models were optimized to simulate net effects on P-gp. Among the tested P-gp induction by enzalutamide, fold-increase in P-gp expression of 1.0 (no induction) resulted in the closest AUC ratio to observed (Table 4, Fig. 3).

### DDI simulation with apixaban and rivaroxaban

The DDI simulation with apixaban and rivaroxaban was performed under the assumption of no P-gp induction effect (P-gp fold increase of 1.0) of enzalutamide and M2 in the models. After single oral administration of 10 mg of apixaban with 160 mg of enzalutamide after 50 days of multiple oral administrations, a 31% decrease in AUC and no change in C_max_ were predicted (Table 5).

**Table 5:** Simulated AUC and C_max_ ratios of apixaban and rivaroxaban in presence to absence of 160 mg multiple dose of enzalutamide

	Apixaban			Rivaroxaban Model 1		Rivaroxaban Model 2	
	AUC_inf_ ratio	C_max_ ratio		AUC_last_ ratio	C_max_ ratio	AUC_last_ ratio	C_max_ ratio
Population simulation							
GMR (90%CI)	0.691 (0.672–0.709)	1.04 (1.02–1.06)		0.552 (0.535–0.571)	0.746 (0.733–0.758)	0.578 (0.556–0.602)	0.767 (0.752–0.781)
Sensitivity analysis							
Intestinal P-gp RAF/REF			Intestinal P-gp RAF/REF				
2.5	0.651	0.916	0.015	0.561	0.711	0.592	0.740
4.0	0.656	0.930	0.024	0.561	0.713	0.593	0.742
10	0.675	0.980	0.060	0.564	0.719	0.595	0.748
25†	0.711	1.07	0.15†	0.569	0.732	0.601	0.761
100	0.815	1.25	0.60	0.586	0.793	0.619	0.801

RAF/REF: relative activity factor/relative expression factor, †: default RAF/REF setting

After single oral administration of 20 mg of rivaroxaban with 160 mg of enzalutamide after 50 days of multiple oral administrations, a 45% and 42% decrease in AUC and a 25% and 23% decrease in C_max_ were simulated in Model 1 (with OAT3 model) and Model 2 (without OAT3 model), respectively. Sensitivity analysis of intestinal P-gp activity on enzalutamide’s effect on apixaban and rivaroxaban exposure was tested. With apixaban, AUC and C_max_ ratios ranged from 0.651 to 0.815 and from 0.916 to 1.25, respectively, for the tested intestinal P-gp RAF/REF of 2.5 to 100 (default 25). With rivaroxaban, AUC and C_max_ ratios ranged from 0.561 to 0.619 and from 0.711 to 0.801, respectively, for the tested intestinal P-gp RAF/REF of 0.015 to 0.60 (default 0.15).

## Discussion

Enzalutamide and M2 PBPK models were developed and verified to be used for predicting DDI with CYP3A substrates and P-gp substrates. The plasma concentration profiles of enzalutamide and M2 were simulated using the developed PBPK models, giving similar profiles to those clinically observed after single and multiple doses. The simulation results well captured the observed plasma concentration profiles of enzalutamide and M2, except for C_max_ of parent after single-dose administration of enzalutamide (Fig. 1, Table S1). The simulated C_max_ of enzalutamide after single-dose administration was 40% lower than observed; however, after multiple-dose simulation, simulated AUC, C_max_, and C_min_ are close to those observed and underestimation in C_max_ after a single dose was considered not to influence the DDI simulation. Although enzalutamide and M2 showed induction effects on CYP2C8 in an in vitro study, a clinical DDI study with a sensitive CYP2C8 substrate, pioglitazone, indicated enzalutamide did not cause a clinically meaningful interaction on CYP2C8 [2], probably because enzalutamide and M2 have the potential to inhibit CYP2C8 and inhibition and induction offset the clinical effect on CYP2C8 substrate exposure. The in vitro determined CYP3A4 induction parameters was calibrated with positive control, rifampicin, and in vitro to in vivo extrapolation of CYP3A4 induction effects were successfully shown in the present analysis.

The prediction of P-gp induction with a PBPK model can be challenging as fundamental physiological information, such as turnover of P-gp in each organ, is sparse and in vitro to in vivo extrapolation of transporter induction has not widely been investigated. As a result, several PBPK works employed a static assumption in P-gp induction by rifampicin [11, 12]. After multiple-dose administration of 600 mg of rifampicin, a 3.5-fold increase in intestinal P-gp expression was determined by Western blot [13], and this value had been used in PBPK analysis of P-gp induction by rifampicin. In contrast, there is a lack of clinical P-gp expression data with other drugs, such as enzalutamide, that have P-gp induction potential in vitro. As enzalutamide showed both P-gp inhibition and induction effects in in vitro studies, a clinical DDI study would only reveal the net effect on P-gp. The clinical net effect of enzalutamide on P-gp was investigated with a typical P-gp substrate, digoxin [3]. Multiple oral administration of 160 mg of enzalutamide increased digoxin AUC and C_max_ by 29% and 17%, respectively, indicating enzalutamide’s inhibition effect on P-gp outweighed its induction effect. In this PBPK model analysis, increase in AUC was reproduced with the assumption of no induction on P-gp and use of in vitro P-gp K_i_ values (Table 4, Fig. 3). Under these assumptions, C_max_ ratio was slightly overpredicted which may be a representation of stronger induction or weaker inhibition of intestinal P-gp in the clinical setting compared to model assumptions. In the clinical study, a decrease in renal clearance of digoxin was observed in the presence of enzalutamide, which may be a result of enzalutamide’s inhibitory effect on renal P-gp, which is not accounted for in the present PBPK analysis [3]. Since inhibition of renal P-gp results in an increase in AUC only, not incorporating this mechanism into the model could be a possible reason for overestimation in the C_max_ ratio. Simulations of P-gp induction scenarios (fold increase in P-gp expression of 2.0 or 3.5) were not able to show an increase in digoxin exposure observed in the clinical study unless P-gp K_i_ values were adjusted from in vitro values (Table 4). A 3.5-fold increase in intestinal P-gp and 5-fold decrease in P-gp K_i_ resulted in similar change in digoxin exposure (data not shown). Lack of intestinal P-gp expression change data after multiple doses of enzalutamide prevents us from selecting the “true scenario”. Instead, we pursued to develop a fit-for-purpose model which can reproduce the net effect on digoxin exposure in presence of enzalutamide.

The developed enzalutamide and M2 PBPK models were applied to the DDI simulation with apixaban and rivaroxaban. The simulation results showed a stronger interaction with rivaroxaban compared to apixaban. This is probably due to two reasons: one is the higher contribution of CYP3A to rivaroxaban elimination than apixaban, and another is the higher contribution of P-gp to intestinal absorption in apixaban than rivaroxaban. In the model development process of apixaban and rivaroxaban, the contribution of CYP3A to hepatic metabolism was assumed to be 42% and 61%, respectively, and these assumptions were verified with results from many clinical DDI studies using CYP3A inhibitors [10]. In the sensitivity analysis of intestinal P-gp activity, the change in rivaroxaban exposure in the presence of enzalutamide was not affected by intestinal P-gp activity, indicating P-gp does not play an important role in rivaroxaban intestinal absorption. In apixaban, change in intestinal P-gp activity has more impact on apixaban exposure when co-administered with enzalutamide compared to rivaroxaban; however, the impact is still not large. In recent publications, the involvement of intestinal P-gp on apixaban and rivaroxaban absorption was investigated using absorption rates in the presence and absence of P-gp inhibitors and inducers as an indicator of P-gp influence in the intestine [14, 15]. The mean absorption time and t_max_ of apixaban and rivaroxaban when concomitantly administered with P-gp inhibitors and inducers suggested limited or absence of effect of efflux transport of apixaban and rivaroxaban by P-gp in the intestine.

For rivaroxaban, two models with different assumptions on renal elimination (low passive permeability and OAT3 involvement or high passive permeability and no OAT3 involvement) were tested. Both enzalutamide and M2 showed OAT3 inhibitory effects in an in vitro study; in vitro K_i_ values of 15.1 and 11.5 µmol/L, respectively, were incorporated into the models. However, DDI simulation results between Model 1 and Model 2 were similar, and inhibition of OAT3 with enzalutamide and M2 did not result in a substantial change in rivaroxaban plasma exposure.

Rifampicin decreased apixaban and rivaroxaban AUC by 54% and 49%, respectively [16, 17]. The interaction with enzalutamide is estimated to be milder in apixaban (i.e. 31% vs 54%) and mild to similar in rivaroxaban (i.e. 45% vs 49%) compared to rifampicin. Rifampicin also has the potential to inhibit and induce P-gp, even though the clinical DDI study with P-gp substrates indicated rifampicin acts as a P-gp inducer [13]. Multiple oral administration of 600 mg of rifampicin decreased midazolam AUC more than 90% (AUC ratio less than 0.1) [18] and rifampicin’s ability to induce CYP3A is considered stronger than that of enzalutamide. Combined with potential net P-gp induction by rifampicin and potential net P-gp inhibition by enzalutamide, the exposure-lowering effect to dual substrates of P-gp and CYP3A should be stronger in rifampicin than enzalutamide. The present PBPK analysis results support this hypothesis. Rifampicin is defined as a combined P-gp and strong CYP3A inducer, and hence concomitant use with apixaban and rivaroxaban should be avoided per prescribing information of apixaban and rivaroxaban. In contrast, enzalutamide is not a combined P-gp and strong CYP3A inducer, and the exposure change in apixaban and rivaroxaban when concomitantly administered with enzalutamide is estimated to be milder than with rifampicin. Enzalutamide will be concomitantly used with these DOACs. However, a decrease in plasma exposure of apixaban and rivaroxaban based on CYP3A induction was suggested, and care should be taken when apixaban and rivaroxaban are concomitantly administered with enzalutamide.

As for alternative anticoagulant therapy using other DOACs such as dabigatran and edoxaban, both of which are known as P-gp substrates [7], enzalutamide should be concomitantly used with following the instruction for P-gp inhibitors in the labels of these drugs because dabigatran and edoxaban are not substrates of CYP3A and enzalutamide is considered to act simply as a net P-gp inhibitor. Enoxaparin, another DOAC, is not a substrate of P-gp, CYP3A, and other enzymes which enzalutamide may affect, such as CYP2C9 and CYP2C19. Interaction between enzalutamide and enoxaparin is not foreseen.

There are several model limitations. First, there is only one clinical DDI data between enzalutamide and P-gp substrates which prevents extensive verification of enzalutamide and M2 PBPK models as net P-gp inhibitor. P-gp inhibition and induction of the models were calibrated only from one clinical study results, although, preferably, the verification should be conducted with several independent clinical datasets. Second, as a result of challenges in extrapolating in vitro P-gp induction data to in vivo, static assumption in P-gp induction simulation was used. Discrepancy between in vitro and in vivo P-gp inhibition constants had also been reported [19]. For the accurate simulation of net effect on P-gp, robust in vitro to in vivo extrapolation of P-gp inhibition and induction, together with dynamic modeling in PBPK, is needed. Third, the absence of clinical DDI data between enzalutamide and apixaban or rivaroxaban means that the accuracy of the predicted DDI cannot be confirmed. However, based on careful verification of apixaban and rivaroxaban as CYP3A and P-gp substrates [10] and clinical evidence of the potential of enzalutamide as a CYP3A inducer and net P-gp inhibitor, the predicted interaction provides reasonable caution regarding concurrent use of these drugs.

## Conclusions

Enzalutamide and M2, a major metabolite of enzalutamide, PBPK models were developed and verified as CYP3A inducers and net P-gp inhibitors by incorporating in vitro determined CYP3A induction data and P-gp inhibition constant to the models. The model predicted possible interaction with apixaban and rivaroxaban when concomitantly administered after 160 mg multiple doses of enzalutamide. Although the predicted decreases in apixaban and rivaroxaban plasma exposure were milder than those observed with rifampicin, concurrent use of these drugs warrants careful monitoring for efficacy and safety.

## Supplementary Information

Below is the link to the electronic supplementary material.

Supplementary file1 (PDF 101 KB)
