# Supplementary material for: Physiologically-based pharmacokinetic modeling to predict drug-drug interaction of enzalutamide with combined P-gp and CYP3A substrates
Source: J Pharmacokinet Pharmacodyn. 2023 Jun 21;50(5):365–76. doi: 10.1007/s10928-023-09867-7 (PMC10460728; doi:10.1007/s10928-023-09867-7)
Supplement: Supplementary file 1 — Supplementary file1 (PDF 101 KB) [file 10928_2023_9867_MOESM1_ESM.pdf]

Physiologically-based pharmacokinetic modeling to predict drug-drug interaction of enzalutamide with combined P-gp and CYP3A substrates

Yukio Otsuka[1], Srinivasu Poondru[2], Peter L. Bonate[2], Rachel H. Rose[3], Masoud Jamei[3], Fumihiko Ushigome[4], Tsuyoshi Minematsu[5]

[1] Clinical Pharmacology and Exploratory Development, Astellas Pharma Inc., Tokyo, Japan

[2] Clinical Pharmacology and Exploratory Development, Astellas Pharma Global Development Inc., IL, USA

[3] Certara UK, Simcyp Division, UK

[4] Applied Research and Operations Astellas Pharma Inc., Ibaraki, Japan

[5] Immuno-oncology, Astellas Pharma Inc., Ibaraki, Japan

Corresponding author: Yukio Otsuka; yukio.otsuka@astellas.com

Table S1. Comparison of pharmacokinetic parameters of enzalutamide and M2 after single oral administration of 160 mg enzalutamide observed in a clinical study and predicted

**Enzalutamide**								
	Observed				Simulated			
	AUC_{inf} (μg·h/mL)	t_{max} (h)	C_{max} (μg/mL)	$t_{1/2}$ (h)	AUC_{inf} (μg·h/mL)	t_{max} (h)	C_{max} (μg/mL)	$t_{1/2}$ (h)
Mean	288	-	5.66	77.7	395	-	3.35	136
SD	69.7	-	0.666	25.34	219	-	0.425	90.33
CV%	24.2	-	11.8	32.6	55.5	-	12.7	66.3
Median	305.6	0.75	5.640	82.54	341.5	1.25	3.339	110.2
Min	164	0.50	4.64	36.0	68.5	0.80	2.00	10.8
Max	383	1.50	6.74	128	1050	2.65	4.58	446
P/O ratio	-	-	-	-	1.37	1.67	0.592	1.75
**M2**								
	Observed				Simulated			
	AUC_{inf} (μg·h/mL)	t_{max} (h)	C_{max} (μg/mL)	$t_{1/2}$ (h)	AUC_{inf} (μg·h/mL)	t_{max} (h)	C_{max} (μg/mL)	$t_{1/2}$ (h)
Mean	395	-	0.887	179	379	-	0.931	160
SD	64.8	-	0.1966	35.2	148	-	0.3490	86.7
CV%	16.4	-	22.2	19.6	39.0	-	37.5	54.3
Median	377	168	0.827	175	348	142	0.920	136
Min	286	72.0	0.70	134	124	51.6	0.29	42.4
Max	513	264	1.32	271	747	419	1.88	492
P/O ratio	-	-	-	-	0.959	0.845	1.05	0.894

Table S2. Comparison of pharmacokinetic parameters of enzalutamide and M2 after once-daily multiple oral administration of 160 mg enzalutamide for 49 days observed in a clinical study and predicted

**Enzalutamide**								
	Observed				Simulated			
	AUC_{tau} (μg·h/mL)	C_{max} (μg/mL)	C_{min} (μg/mL)	PTR	AUC_{tau} (μg·h/mL)	C_{max} (μg/mL)	C_{min} (μg/mL)	PTR
Mean	321.5	16.59	12.00	1.266	311.3	14.92	11.83	1.366
SD	85.39	3.812	3.512	0.1271	158.4	6.716	6.496	0.3286
CV%	26.6	23.0	29.3	10.0	50.9	45.0	54.9	24.1
Median	295.6	15.55	11.45	1.240	288.0	13.93	10.59	1.293
Min	240	11.8	6.92	1.09	36.0	3.19	0.789	1.09
Max	593	28.0	22.4	1.51	927	39.5	36.3	4.04
P/O ratio	-	-	-	-	0.968	0.899	0.986	1.08

**M2**								
	Observed				Simulated			
	AUC_{tau} (μg·h/mL)	C_{max} (μg/mL)	C_{min} (μg/mL)	MPR (MWC)	AUC_{tau} (μg·h/mL)	C_{max} (μg/mL)	C_{min} (μg/mL)	MPR (MWC)
Mean	278.3	12.68	10.57	0.913	318.6	13.34	13.25	1.27
SD	85.47	3.773	3.271	0.2817	123.9	5.211	5.221	0.7869
CV%	30.7	29.7	30.9	30.8	38.9	39.0	39.4	61.9
Median	263.2	12.10	10.23	0.886	306.4	12.78	12.70	1.10
Min	182	8.65	6.99	0.56	90.2	3.79	3.65	0.37
Max	442	19.6	16.5	1.66	672	28.1	28.0	5.72
P/O ratio	-	-	-	-	1.14	1.05	1.25	1.39
